# Variation in Linseed Oil Composition: Impact of Cultivar, Cultivation System, and Year of Cultivation

**DOI:** 10.3390/molecules30040875

**Published:** 2025-02-14

**Authors:** Grzegorz Dąbrowski, Małgorzata Tańska, Sylwester Czaplicki, Tadeusz Sadowski, Bogumił Rychcik, Marta K. Kostrzewska, Zofia Antoszkiewicz, Iwona Konopka

**Affiliations:** 1 Department of Food Plant Chemistry and Processing, Faculty of Food Sciences, University of Warmia and Mazury in Olsztyn, Plac Cieszyński 1, 10-726 Olsztyn, Poland; grzegorz.dabrowski@uwm.edu.pl (G.D.); sylwester.czaplicki@uwm.edu.pl (S.C.); iwona.konopka@uwm.edu.pl (I.K.); 2 Department of Agroecosystems and Horticulture, Faculty of Agriculture and Forestry, University of Warmia and Mazury in Olsztyn, Plac Łódzki 3, 10-718 Olsztyn, Poland; tadeusz.sadowski@uwm.edu.pl (T.S.); bogumil.rychcik@uwm.edu.pl (B.R.); marta.kostrzewska@uwm.edu.pl (M.K.K.); 3 Department of Animal Nutrition and Feed Science, Faculty of Animal Bioengineering, University of Warmia and Mazury in Olsztyn, Oczapowskiego 5, 10-719 Olsztyn, Poland; zofia.antoszkiewicz@uwm.edu.pl

**Keywords:** linseed, fatty acids, sterols, tocopherols, carotenoids, phenolics, crop rotation, herbicide

## Abstract

Linseed oil quality depends on cultivar and agriculture practice/conditions. In this study, we analyzed the effect of the main variable, which was continuous cropping system vs. crop rotation system. The aim of this study was to compare the oil content and composition (fatty acid composition and sterol, tocochromanol, carotenoid, and phenolic contents) of two fiber-type linseed cultivars (Modran and Nike). All measurements were performed according to standard chromatographic/spectrophotometric procedures typical for oil analysis. The factors that affected the crop yield and oil quality of the tested cultivars included the cultivation system (crop rotation or continuous cropping), plant protection level (herbicide application or no herbicide application), and year of cultivation (2018 and 2019). The cultivars exhibited high oil content (35.4–42.7%) with substantial omega-3 fatty acid (α-linolenic acid) content (53.6–62.2% of total fatty acids). The primary bioactive components in all the oils were sterols (dominated by β-sitosterol), with their content reaching 5079 mg/kg, and tocochromanols, with their content reaching 679 mg/kg (dominated by γ-tocopherol), which was influenced by all of the studied factors. In contrast, carotenoids and phenolic compounds constituted a smaller fraction of the oils (up to 17 and 159 mg/kg, respectively), but their content was the most variable and was strongly dependent on the cultivation year and cultivation system (CV 21 and 37%, respectively). In summary, the results of the current study showed that continuous linseed cropping resulted in increased levels of carotenoids, phenolic compounds, and γ-tocopherol in oil. Our findings indicated that the oil content was mostly affected by the cultivar and cultivation year, while the α-linolenic acid content in the oil was also affected by plant protection practices. These findings may be helpful in predicting the composition of obtained linseed oil and applying proper cultivation technology, depending on the purpose of oil usage.

## 1. Introduction

Linseed, also known as flaxseed (*Linum usitatissimum* L.), is an annual plant cultivated in three primary types: oil, oil–fiber, and fiber [1]. As of 2022, linseed was cultivated in over 50 countries worldwide, with France, Belgium, Belarus, China, and the Russian Federation among the major producers [2]. For example, in 2022, France recorded the highest production of fiber-type linseed (raw or retted) worldwide, at 652,680 tons. The same source reported that total oil-type linseed production was 3,973,932 tons of seeds, with the main producers being the Russian Federation (44%), Kazakhstan (21%), Canada (12%), mainland China (7%), and India (3%).

The food industry is particularly interested in linseed due to its valuable chemical composition. Linseed consumption offers potential health benefits, including reductions in arthritis, atherosclerosis, cancer, cardiovascular disease, diabetes, osteoporosis, and autoimmune and neurological disorders. More data are available in the review by Al-Madhagy et al. [3], which discusses mechanisms related to the phytochemical composition of linseed oil, highlighting its antioxidant, anticancer, anti-osteoporosis, anti-inflammatory, and antibacterial activities.

Among the main components of linseed, oil has attracted the greatest scientific interest in recent years [4]. Linseed typically contains approximately 40% oil, which primarily includes α-linolenic acid (ALA), an omega-3 (n-3) fatty acid recommended in the diet. This fatty acid acts as a precursor to essential long-chain unsaturated fatty acids, such as eicosapentaenoic acid (EPA) and docosahexaenoic acid (DHA) [5]. For people who avoid fish and seafood products, ALA-rich plant oils are an excellent choice for their daily diet. Research has indicated that linseed and chia seeds are two plants with the highest content of ALA in oil [6,7,8].

A study conducted in India with 48 linseed genotypes showed that the oil content in the analyzed samples ranged from 34.0% to 42.3%, with ALA accounting for 33.1% to 54.8% of the oil [9]. Similarly, the oil content in the seeds of 84 linseed genotypes cultivated in Poland was in the range of 40.7–44.8%, with the ALA content ranging from 48.4% to 58.9% [10]. Another study of 81 linseed genotypes originating from Austria, Bulgaria, the Czech Republic, Germany, Hungary, Russia, the Slovak Republic, Sweden, and Turkey, cultivated in experimental fields at Ankara University (Turkey), gave a crop yield with a medium oil content of 29.75%, with a mean ALA content of 53.46% [11]. Among the cited studies, only Silska and Walkowiak [10] compared linseed type (oil, fiber, and combined), and their results showed that the ALA content in fiber-type linseed can be as high as that in oil-type linseed. The presented data additionally suggest a relatively stable proportion of ALA in linseed oil, with climate being a decisive factor. Studies conducted in Finland have shown that cooler climates in temperate regions can increase the ALA content to over 60% [12]. Similar results were found for linseed from New Zealand and Canada, where ALA content reached 60%. This high content was credited to the cool and humid climates of these regions [13]. Such dependence was also confirmed in a study performed by Rossi et al. [14], where lower temperature and more rainfall were related to higher contents of ALA. Similarly, the significant influence of climate conditions was presented by Čeh et al. [15].

The yield of linen seed is influenced by cultivar selection and cultivation practices. Linseed can be cultivated organically or under no-till regimes, and it can be grown with relatively low input depending on the soil type and precipitation levels [16]. However, recent studies conducted in Romania showed that rainfall in late May–June is a key factor for obtaining improved cultivar efficiencies, and that high temperatures and low rainfall in July and August are beneficial for crop yield and quality [17]. Another study showed that water shortage and wet and cold soil in spring and high temperatures in summer are factors that negatively affect seed yield [15]. It is worth emphasizing that linseed cultivation exemplifies the goals of recent European Union agricultural policies regarding protection against climate change, such as the Common Agricultural Policy and the European Green Deal [18].

Two main plant cultivation systems used in agriculture are continuous cultivation and crop rotation. In general, most crops react negatively to continuous cultivation with such unfavorable phenomena as excessive growth of weed infestation; the development of fungal diseases in the root system, stem base, leaves, and spikes; and the multiplication of specialized pests in the soil (more data available in Jastrzębska et al. [19]). Comparisons of the main cultivation systems have been made possible through conducting long-term field experiments. The oldest worldwide experiments have been conducted in Rathamstad (UK) since 1843 (continuous winter wheat cultivation) and since 1852 (continuous spring barley cultivation) [20]. To the best of our knowledge, there is no long-term agricultural research on linseed cultivation outside Poland. However, long-term agricultural experiments on major crops have been conducted in Poland since 1967. Fiber linseed has been cultivated in continuous cropping since 1968, and in crop rotation since 1973 [19]. In these experiments, the typical agricultural characteristics of the cereals, such as grain yield, spike density, grains per spike, weight of 1000 grains, and weed biomass, are analyzed [19,21]. 

To the best of our knowledge, no studies have analyzed the effect of cropping systems (crop rotation vs. continuous cropping) or crop protection practices on oil content and composition. Thus, in the present study, we focused on how these agronomic factors influence not only the oil yield but also fatty acid composition and the profiles of key bioactive compounds, such as sterols, tocochromanols, carotenoids, and phenolics in two popular and frequently harvested fiber-type linseed cultivars registered in Poland (Modran and Nike). The oil content and quality (composition) were evaluated by varying the following conditions:

1. Cultivation system: crop rotation (potato, oat, fiber linseed, winter rye, faba bean, winter triticale) vs. monoculture (continuous linseed cropping);
2. Plant protection level: herbicide application (+) vs. no herbicide protection (−);
3. Cultivation year: 2018 vs. 2019 (varied by precipitation and daily air temperature).

This focus on agronomic practices provides valuable insights for optimizing linseed cultivation based on specific production goals, which distinguishes our work from the previously published research.

## 2. Results and Discussion

### 2.1. Oil Content

The oil content of the analyzed linseed samples varied from 35.4% to 42.7% (Table 1). The most significant factor influencing this variable was the “year” of cultivation (Table S1). The effects of “cultivar” and some interactions (year*herbicide, year*cultivar*crop rotation, cultivar*herbicide*crop rotation, and year*cultivar*herbicide*crop rotation) also exceeded statistical significance at *p* ≤ 0.05, but their impact was weaker compared to the effect of “year” (Table S1).

Filipovic et al. [22] also found statistically significant variations in linseed oil content depending on the cultivation year. They concluded that the year of cultivation, soil type, and meteorological conditions during the growing season all affect oil accumulation in linseeds. The initial development stage (flowering and early seed development, just prior to seed ripening) was crucial for linseed; drought during this stage can significantly impair seed development and oil content. Maximization of oil yield can be achieved by maintaining adequate soil moisture during the corresponding periods [15]. In our study, April and May of 2018 were denoted as dry months (with only April being dry in 2019), which confirms a phenomenon observed by the cited authors. The influence of the crop year was also confirmed by Čeh et al. [15], who demonstrated that the seed oil content was significantly higher in 2013 compared to 2012. Their study attributed the unusual 2013 harvest conditions to wet and cold soil in spring, followed by a prolonged summer drought accompanied by high temperatures. They suggested that these conditions led to a lower seed yield but, conversely, an increased oil content. However, they found no significant differences in oil content among the different cultivars. In contrast, Jarošová et al. [23] reported differences in oil content between some linseed cultivars grown in the Czech Republic. The influence of genotype was associated with seed color, with brown-seeded cultivars exhibiting significantly higher oil content compared to yellow-seeded cultivars. Additionally, they observed that a lack of precipitation in the spring (May and June in Central Europe) of 2019 resulted in the lowest oil content in most cultivars, along with a negative impact on seed yield.

Based on the presented literature data, our study confirms the significant influence of cultivar and climatic conditions on the oil content of fiber-type linseeds. However, no significant effect of the cultivation method (continuous cropping vs. crop rotation) was observed.

### 2.2. Fatty Acid Composition

The fatty acid composition of the analyzed oils is presented in Table 1. The main representative was ALA with a content from 53.6% to 62.2% of all fatty acids. This was followed, in decreasing order, by oleic (14.1–21.2%), linoleic (14.6–17.5%), palmitic (4.31–4.92%), stearic (2.32–4.81%), and vaccenic acid (below 1%). Generally, such fatty acid composition is typical for high-linolenic linseed oil [24,25]. However, the observed fluctuations in fatty acid composition could be attributed to the influence of the analyzed variables. The results of the variance analysis are provided in Table S1. In the case of ALA, its content was mainly affected by “year” and “cultivar”. The use of “herbicide” also showed statistical significance at *p* ≤ 0.05, but its effect was much weaker compared to the previously mentioned factors.

These dependencies are well documented in the literature. For example, in a study presented by Trela et al. [26], the content of ALA varied from 44% to 59%, depending on linseed genotype. Similar to our findings, the cited study also found a higher ALA content in the “Modran” cultivar compared to the “Nike” cultivar. Walkowiak et al. [27] further confirmed the significant influence of genotype and cultivation year on the ALA content in linseed oils. An ALA content of at least 55% of total fat is considered optimal for edible linseed oil production [28]. Samples from continental and temperate climates had higher contents of ALA (polyunsaturated) and lower contents of oleic acid (monounsaturated) than samples from Mediterranean and subtropical climates [28]. Similar conclusions were presented in other studies [12,13]. Poland is a country with a temperate climate, which may favor ALA biosynthesis. In turn, Andruszczak et al. [29] found that increased mineral fertilization (up to 80 kg N/ha) combined with intensive crop protection against weeds significantly increased the content of ALA in Szafir and Oliwin linseed cultivars. This corresponds with the findings of our study, where higher ALA content in linseed oil was related to the application of herbicide. The main factor (crop rotation system) investigated in the experiment had little effect on ALA content.

### 2.3. Sterol Content

**Figure 1:**
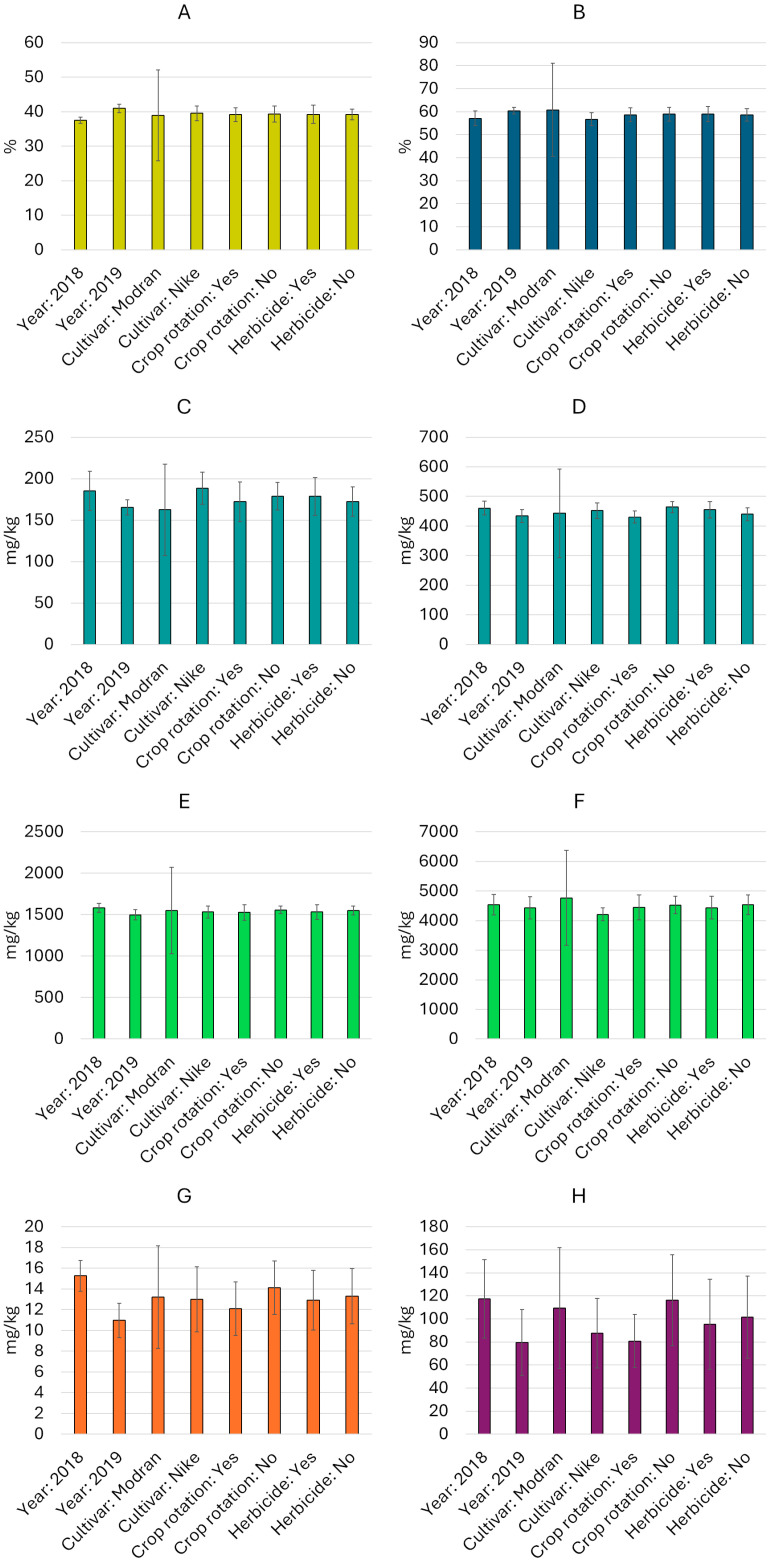
Average contents (mean value and standard deviation) of oil and selected bioactive compounds by experimental variable ((**A**)—oil content, (**B**)—ALA, (**C**)—plastochromanol-8, (**D**)—γ-tocopherol, (**E**)—β-sitosterol, (**F**)—total sterols, (**G**)—total carotenoids, (**H**)—total phenolics).

The total sterol content varied from 3720 mg/kg (cultivar Nike, 2019, crop rotation, with herbicide use) to 5079 mg/kg (cultivar Modran, 2018, crop rotation, without herbicide use) (Table 2). Among the sterols, β-sitosterol prevailed, and its share of the total sterol fraction varied from 31.5% to 37.0%. Following this, the most commonly identified components were cycloartenol (25.9–31.2%), campesterol (15.6–18.2%), and isofucosterol (8.5–11.8%). Stigmasterol and 24-methylene-cyclolanostanol were also found in the analyzed oils. An analysis of variance showed that all the main factors (cultivar, year, crop rotation, and herbicide use) had a significant effect (*p* < 0.05) on the total sterol content, while the β-sitosterol content was only significantly influenced by “year” and “crop rotation” (Table S1). Various interactions were also found among the main factors, which significantly affected both the total sterol and β-sitosterol contents (Table S1). It is worth nothing that the content of the sterols in the Nike cultivar was relatively stable, with an average coefficient of variation (CV) of 5%. In contrast, the sterol content in the Modran cultivar was much more variable, with an average CV of 33% (Figure 1).

Similar sterol contents and compositions in linseed oil were obtained by Ciftci et al. [30]. They found a content of 4072 mg/kg, with β-sitosterol as the main component (35.6% of the total sterols). In another study, the total amounts of sterols in linseed oil were found to be 0.2–0.3%, with β-sitosterol representing more than 55.0%, followed by campesterol (13.1–26.1%) and stigmasterol (3.4–15.0%) [31]. According to Gandova et al. [32], linseed cultivated in Bulgaria contains 0.5% sterols in its oil (a similar value to that found in our study), but the sterol composition differed: β-sitosterol represented 75.5%, stigmasterol 7.2%, brassicasterol 5.9%, and campesterol 5.9%. Herchi et al. [33] found that sterol content in developing seeds is highest near the flowering stage, decreases rapidly up to 14 days after flowering, and then stabilizes at levels ranging from approximately 3000 mg/kg to 5000 mg/kg until seed harvesting.

The results of our study were the first to characterize the composition and sterol content of fiber-type linseed oil while accounting for factors beyond varietal differences. The results demonstrated that the year of cultivation and the crop rotation system exerted a predominant influence on these oil constituents.

### 2.4. Tocochromanol Content

Tocochromanols were the second-most abundant group of bioactive compounds in the analyzed linseed oils, with total content ranging from 564 to 690 mg/kg of oil (Table 3). Across all samples, only two tocochromanol derivatives were identified: γ-tocopherol, comprising 68–78% of the total, and plastochromanol-8, contributing 22–32%. The lowest γ-tocopherol concentration (408 mg/kg) was observed in oil from the Nike cultivar (2019) when neither crop rotation nor herbicide treatment was applied. Conversely, the highest γ-tocopherol content (488 mg/kg) was recorded in oil from the Modran cultivar (2018) under herbicide treatment without crop rotation. Plastochromanol-8 levels appeared to be cultivar-dependent, with generally lower concentrations in Modran oil samples (156–172 mg/kg). In contrast, oils from the Nike cultivar contained up to 219 mg/kg of plastochromanol-8, with the highest levels found in oils from seeds cultivated with both herbicide application and crop rotation.

The analysis of variance showed that all main factors and most interactions significantly influenced the content of both tocochromanols, γ-tocopherol and plastochromanol-8 (*p* < 0.05, Table S1). Among the analyzed factors, the cultivars exhibited greater differentiation (Figure 1): the Nike cultivar showed relatively stable tocochromanol levels (CV up to 10.4%), while the Modran cultivar displayed more variable concentrations of this compound (CV > 33%). Furthermore, plastochromanol-8 showed higher variability across linseed oil samples (except in the Modran cultivar samples), whereas γ-tocopherol content was more stable (CV of 5.5–13.8% and 4.1–6.1%, respectively) (Table 2).

Previous analyses by Trela et al. [26] assessed tocopherol content in the Nike and Modran cultivars. Their findings indicated comparable tocopherol levels in both cultivars, with concentrations close to 180 mg/kg of oil. Gębarowski et al. [34] quantified the total content of tocochromanol-related compounds in cold-pressed oil from the Nike cultivar, reporting a concentration of 355 mg/kg. In this composition, γ-tocopherol was the predominant compound, accounting for 331 mg/kg of oil. Additionally, plastochromanol-8 content in this oil measured 108 mg/kg, while other tocopherol homologues, including α- and δ-tocopherols, constituted approximately 24 mg/kg. Obranović et al. [35] analyzed oils obtained from four cultivars (Altess, Biltstar, Niagara, and Oliwin) cultivated in Zagreb (Croatia), reporting a higher tocochromanol content ranging from 732 to 951 mg/kg of oil. Their findings highlighted a significant influence of climatic conditions during seed development on tocochromanol levels. An increase in temperature and sunshine was associated with higher γ-tocopherol and plastochromanol-8 levels. These results are in line with the present study, where oil samples from seeds harvested in 2018, which experienced generally higher temperatures during seed development, had a higher content of both tocochromanols compared to samples from 2019. Hasiewicz-Derkacz et al. [36] also found higher contents of tocochromanols (approximately 860 mg/kg) in oil from the Linola linseed cultivar cultivated in Poland. Interestingly, the levels of plastochromanol-8 and γ-tocopherol in the oil obtained in their study were comparable.

The results of this study confirm that the content and composition of tocochromanols in linseed oil are influenced by genetic, climatic, and agronomic factors.

### 2.5. Carotenoid Content

Carotenoids were detected in all linseed oils at low concentrations (Table 3), ranging from 9 mg/kg (Nike cultivar, 2019, with herbicide use and crop rotation) to 17 mg/kg (Nike cultivar, 2018, with and without herbicide use and without crop rotation). Overall, the oils from seeds harvested in 2018 contained slightly higher levels of carotenoids than those from 2019 (Figure 1). Additionally, the use of monoculture as a cultivation system appeared to support slightly higher carotenoid biosynthesis. No significant differences in carotenoid concentrations were observed between cultivars or between plant protection systems. The analysis of variance (Table S1) confirmed that these factors, both individually and in combination, had no statistically significant effects (*p* > 0.5). There was also no significant interaction effect between year and these factors separately, nor between crop rotation and the combined factors. Variability analysis within individual variables revealed greater variation for carotenoids than for previously discussed oil components, with oil samples from the Modran cultivar showing the highest variability (CV = 37.3%) (Figure 1). 

Gębarowski et al. [34] reported nearly double the total carotenoid content in oil from the Nike cultivar, with a concentration of 25.03 mg/kg. They also characterized the carotenoid profile in their study, identifying five carotenoids: all-trans-β-carotene, all-trans-lutein, all-trans-zeaxanthin, all-trans-neoxanthin, and all-trans-β-cryptoxanthin. Among these, all-trans-lutein and all-trans-zeaxanthin were the predominant carotenoids, together comprising over 75% of the total carotenoid content. Tavarini et al. [37] also identified lutein as the predominant carotenoid in linseeds. Furthermore, they confirm a strong cultivar dependence on the content of these bioactive compounds in seeds. In contrast, Obranović et al. [35] reported a significantly lower carotenoid content in linseed oil from Croatia, ranging from 1.39 to 2.40 mg/kg. Notably, their findings demonstrated that higher temperatures and reduced rainfall were associated with an increase in carotenoid levels, which is consistent with the results of our study. The cited authors emphasized that carotenoids accumulated in plant seeds play a crucial role in protecting triacylglycerols, unsaturated lipids, membranes, and phenolic quinones from photo-oxidation, which may explain the increased synthesis of carotenoids in developing seeds during periods of higher sunlight exposure.

The results obtained confirm the significant influence of climatic conditions during cultivation on the content of carotenoids in linseed oil.

### 2.6. Phenolic Content

Phenolic compounds were identified as the most variable components in linseed oil, with a CV of 37% across all samples. Total concentrations ranged from 58 mg/kg to 159 mg/kg in the analyzed oils (Table 3). The highest concentrations (>150 mg/kg) of phenolic compounds were observed in oils from Modran seeds harvested in 2018, when monoculture was employed in linseed cultivation, regardless of the plant protection method applied. In contrast, the lowest concentrations of phenolic compounds (<60 mg/100 g) were found in oils from both cultivars grown in 2019 with crop rotation and without herbicide application. An analysis of variance revealed no significant effect of plant protection method (*p* > 0.05) on phenolic compound concentrations in linseed oil. However, other factors significantly influenced these levels (*p* < 0.05, Table S1). Interaction effects between factors were generally insignificant for phenolic compound content, except for the year*herbicide*crop rotation interaction, which had a significant impact (*p* < 0.05). Similarly to carotenoids, phenolic compounds displayed substantial variability within each variable, with CVs ranging from 29% for oil samples from the 2018 crop with crop rotation as the cultivation system, to 47.9% for oil samples from seeds of the Modran cultivar (Figure 1). 

In the study conducted by Gębarowski et al. [34], the total phenolic compound content in oil from the seeds of the Nike cultivar was significantly lower, measuring 238.34 µg/kg of oil, likely due to the cold-pressing extraction method employed. Among the phenolic compounds identified, vanillin was present at the highest concentration (95.40 µg/kg), followed by ferulic acid (48.66 µg/kg) and vanillic acid (32.78 µg/kg). Additionally, p-coumaric acid and coniferyl aldehyde were quantified at 20.98 µg/kg and 23.35 µg/kg, respectively. Other identified phenolic compounds, such as syringaldehyde, o-coumaric acid, and secoisolariciresinol, were present at levels below 8 µg/kg. The significant effect of cultivar on the accumulation of phenolic compounds in linseeds was demonstrated by Tavarini et al. [37], Herchi et al. [38], and Özcan and Uslu [39]. In addition, Herchi et al. [38] highlighted that the phenolic compound content in linseed oil is strongly influenced by the maturity stage of the seeds. Their study revealed that, during the early stages of development (7 days after flowering, DAF), the total phenolic content in oil from three linseed cultivars ranged from 140.71 to 107.14 mg caffeic acid equivalent (CAE)/kg of oil. However, this content decreased progressively during seed maturation, reaching significantly lower levels of 14.23–16.64 mg CAE/kg of oil at harvest (56 DAF). The authors explained this phenomenon by changes in the chemical composition of the lipid fraction, which likely altered the solubility of phenolic compounds. Ferulic acid and vanillic acid were identified as the predominant phenolic acids in the oil, while vanillin was the main simple phenol. Interestingly, the variation in phenolic compound content among different cultivars was significantly lower in oils extracted from mature seeds compared to those from seeds at earlier developmental stages. It is worth noting that a higher content of phenolic compounds in oils may positively influence oxidative stability. Furthermore, these compounds have been reported to act as stabilizers of tocopherols, which in turn can protect and regenerate carotenoids [36].

The results of our study showed the greatest significant influence of factors such as year, crop rotation, and year*herbicide*crop rotation on the phenolics content of linseed oil.

### 2.7. Principal Component Analysis and Correlation Analysis

**Figure 2:**
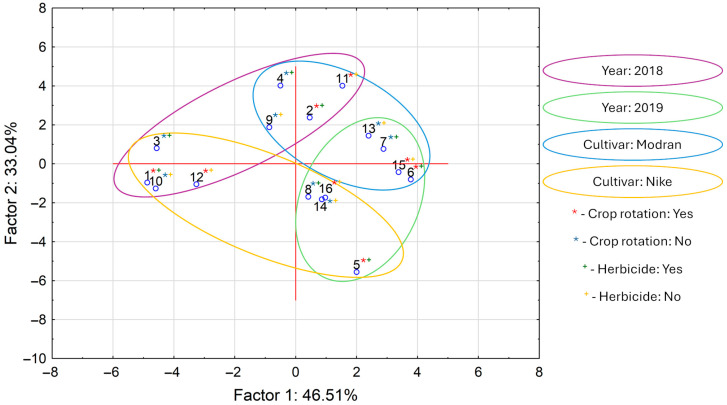
Score plot for principal component analysis (PCA) applied to all determined variables in linseed oils (numbers in plot corresponds in sample numbers in all tables).

The graphic interpretation of PCA calculations for all analyzed variables is presented in Figure 2. Interpretation of these data revealed that the first two main components, PC1 and PC2, explain almost 80% of the total variance. The score plot clearly reflects differences between the samples that differed across the variables of “year”, “cultivar”, “crop rotation”, and “herbicide”. On the upper left side of the plot are samples cultivated in 2018. The lower right side of the graph shows the samples cultivated in 2019. Similarly, on the lower left side are samples from the Nike cultivar, and on the upper right side are samples from the Modran cultivar. The variables “crop rotation” and “herbicide” did not separate the samples into visible groups on the score plot. In summary, the PCA results showed that “cultivar” and “year” were the most decisive for overall oil composition.

The results of the correlation analysis between the studied variables, presented in Table 4, revealed a significant (*p* ≤ 0.05) positive correlation between oil content and linoleic acid (r = 0.89). In contrast, significant (*p* ≤ 0.05) negative correlations were observed with oleic acid, campesterol, stigmasterol, β-sitosterol, total carotenoids, total phenols, γ-tocopherol, and total tocochromanols (r ranging from −0.54 to −0.70). Moreover, significant correlations (*p* ≤ 0.05) were identified between fatty acids and bioactive compounds in the oils. Palmitic, stearic, oleic, and vaccenic acids were positively correlated with total carotenoids (with the exception of stearic and vaccenic acids), plastochromanol-8, and total tocochromanols (r ranging from 0.65 to 0.91). Negative correlations were observed with isofucosterol and cycloartenol (r ranging from −0.89 to −0.56). Additionally, palmitic acid was positively correlated with stigmasterol (r = 0.61), while stearic acid exhibited a negative correlation with 24-methylene-cyclolanostanol. Linoleic acid was negatively correlated with major sterols, γ-tocopherol, total carotenoids, phenols, and tocochromanols (r ranging from −0.52 to −0.87). Conversely, ALA was positively associated with isofucosterol, cycloartenol, and total sterols (r > 0.54) but negatively correlated with plastochromanol-8, as well as total tocochromanols and carotenoids (r ranging from −0.51 to −0.91). Significant correlations (*p* ≤ 0.05) were also observed among bioactive components. Total carotenoids were positively correlated with major sterols, total phenols, and both total and individual tocochromanols (r ranging from 0.52 to 0.70). Total phenols were positively correlated with campesterol, β-sitosterol, and total sterols, with an average correlation coefficient of r = 0.55.

Obranović et al. [35], while studying the influence of climate, cultivar, and production process on linseed oil composition, also confirmed a positive correlation between total carotenoids and plastochromanol-8 and total tocochromanols, similar to our findings. The correlation analysis for linseed oil conducted by Zhang et al. [40] showed that total oil content was highly negatively correlated with total phenols, carotenoids, stigmasterol, and α-tocopherol. At the same time, the ALA content was negatively affected by palmitic, stearic, and oleic acid content. In contrast to our results, the ALA content was negatively correlated with total sterols, mostly 24-methylene cycloartenol and campesterol. As can be seen, the relationships identified are not all consistent, highlighting the need for further research to better understand the mechanisms of compound synthesis in the seeds and their transfer to the oil.

## 3. Materials and Methods

### 3.1. Plant Material

Linseed (Institute of Natural Fibers and Medicinal Plants, Poznań, Poland) was cultivated at the Production and Experimental Plant “Bałcyny” LLC in Bałcyny (Poland, Warmian-Masurian Voivodeship, 53.35° N, 19.51° E). Detailed information on soil properties can be found in the study by Kostrzewska and Jastrzębska [21], while the precipitations and temperatures during the growing seasons are shown in Table 5. Linseed was sown in April and harvested in August for both years. The mineral fertilization applied to all plots was 200 kg NPK (N–40 kg/ha, P_2_O_5_–60 kg/ha, and K_2_O–100 kg/ha), and the herbicides used were Chisel 51.6 WG (DuPont, Wilmington, USA) in 2018 and Chwastox Extra 300 SL (Ciech, Nowa Sarzyna, Poland) in 2019. The field experiment was conducted in triplicate. After harvesting, the seeds were manually cleaned, dried to a moisture content below 9% at 40 °C, and stored in a refrigerator (4 °C) until analysis.

### 3.2. Solvents and Reagents

Analytical-grade solvents and reagents, including anhydrous sodium sulfate, chloroform, n-hexane, methanol, sulfuric acid, diethyl ether, ethanol (99.9% purity), potassium hydroxide, and zinc powder, were purchased from Chempur (Piekary Śląskie, Poland). Chromatographic-grade solvents and reagents, including silylating agents, such as pyridine (anhydrous, 99.8% purity, and N,O-bis(trimethylsilyl)trifluoroacetamide with 1% trimethylchlorosilane), n-hexane (≥95% purity), heptane (≥99% purity), and isopropanol (99.5% purity), and standards, such as α-tocopherol (≥96% purity), γ-tocopherol (≥96% purity), δ-tocopherol (≥96% purity), (≥98% purity), and 5-α-cholestane (≥97% purity), were purchased from Sigma-Aldrich (Poznań, Poland). Helium for gas chromatography (99.999% purity) was purchased from Eurogaz-Bombi (Olsztyn, Poland). Deionized water was prepared using an HLP 5 deionizer (Hydrolab, Gdańsk, Poland).

### 3.3. Oil Extraction

Linseeds were conditioned for 48 h at approximately 20 °C and 65% relative humidity to equalize the moisture content across all the samples. The seeds were then ground using an IKA-Werke A20 laboratory mill (Staufen, Germany) under controlled conditions (temperature 20 °C) for a total of 4 min, with a 1 min pause to prevent material overheating. The ground material was weighed to Soxhlet thimbles and extracted with n-hexane in a Soxhlet apparatus according to the Polish Standard PN-EN ISO 659:2010 [41]. Solvent was removed from the extract using a Büchi R-210 rotary vacuum evaporator (Flawil, Switzerland). The oil content was determined based on the mass of oil obtained from the seed weighed. The extracted oils were subsequently used for all further analysis.

### 3.4. Analysis of Fatty Acid Composition

Fatty acid methyl esters (FAMEs) were prepared from extracted oils according to the method described by Dąbrowski et al. [42]. Briefly, 0.02 g of oil was weighed, and 2 mL of a chloroform/methanol/sulfuric acid mixture (100:100:1, *v*/*v*/*v*) was added. The samples were sealed and heated at 70 °C for 2 h to complete methylation. Sulfuric acid was then neutralized by adding zinc powder, and the solvents were evaporated under a nitrogen stream. The remaining FAMEs were dissolved in n-hexane (GC-MS purity) and injected into a GC-MS QP2010 PLUS (Shimadzu, Kyoto, Japan) equipped with a BPX70 (25 m × 0.22 mm × 0.25 μm) capillary column (SGE Analytical Science, Victoria, Australia). The chromatographic separation parameters were as described by Dąbrowski et al. [42]. Helium was used as the carrier gas at a flow rate of 1.3 mL/min. The column temperature program was set as follows: a subsequent increase from 150 °C to 180 °C at a rate of 10 °C/min, then to 185 °C at a rate of 1.5 °C/min, and to 250 °C at a rate of 30 °C/min, followed by a 10 min hold. The GC-MS interface and ion source temperatures were set to 240 °C, and the electron energy was 70 eV. Total ion current (TIC) mode was used over a 50–500 *m*/*z* range.

### 3.5. Analysis of Tocochromanols

The content of tocochromanols was analyzed using high-performance liquid chromatography with fluorescence detection (HPLC-FLD) as described by Dąbrowski et al. [42]. Briefly, 1% (*m*/*v*) oil solutions in n-hexane (HPLC purity) were prepared and injected into an Agilent Technologies (Palo Alto, CA, USA) 1200 series liquid chromatograph equipped with a Reprospher Si 100 (200 mm × 3 mm, 3 μm) column (Dr. Maisch-GmbH, Ammerbuch-Entringen, Germany) and a fluorescence detector set at 296 nm for excitation and 330 nm for emission. Chromatographic separation was performed at 25 °C with a mobile-phase isocratic flow rate of 1 mL/min, using a 0.7% isopropanol solution in n-hexane (*v*/*v*). Retention times for peaks identification were established using standards of α-, β-, γ-, and δ-tocopherol (Merck, Darmstadt, Germany), which were also used to prepare external calibration curves for quantification.

### 3.6. Analysis of Sterols

An amount of 0.2 g of extracted oil combined with 0.2 mL of 5α-cholestane internal standard solution (0.8 mg/mL) was saponified following the method described by Dąbrowski et al. [42]. Unsaponifiable compounds were then extracted with diethyl ether, evaporated to dryness, re-dissolved in 1.5 mL of n-hexane (GC-MS purity), transferred into 1.5 mL glass chromatographic vials, and evaporated to dryness under a nitrogen stream. Next, 100 μL of pyridine and 100 μL of BSTFA (N,O-bis (trimethylsilyl) trifluoroacetamide) with 1% TMCS (trimethylchlorosilane) were added, and the closed sample was left at 60 °C for 60 min to complete derivatization. After silylation, 0.5 mL of heptane (GC-MS purity) was added, and sample was injected into the gas chromatograph equipped with a ZB-5MSi (Phenomenex Inc., Torrance, CA, USA) capillary column. Helium was used as the carrier gas at a flow rate of 0.9 mL/min. The injector temperature was set to 230 °C, and the column temperature program was as follows: 70 °C for 2 min, then increased to 230 °C at a rate of 15 °C/min, then to 310 °C at a rate of 3 °C/min, and then a 10 min hold. The GC-MS interface temperature was 240 °C, and the ion source parameters were set to 220 °C and 70 eV electron energy. The TIC mode was used for quantification in the 100–600 *m*/*z* range. Quantification was based on the internal standard method.

### 3.7. Analysis of Total Carotenoids

The content of carotenoids in oils was determined using the spectrophotometric method described by Toro-Vazquez [43]. Oil solutions in hexane at a concentration of 2.5% (*m*/*v*) were centrifuged in a 5417R centrifuge (Eppendorf Hamburg, Germany) at 1600 rpm for 10 min. Absorbance was measured with a FLUOstar Omega apparatus (BMG Labtech GmbH, Ortenberg, Germany) at a wavelength of 417 nm.

### 3.8. Total Phenolic Content

The total phenolic content was determined using the spectrophotometric method with Folin–Ciocalteu reagent as described by Siger et al. [44]. An amount of 0.5 g of oil was weighed into a 2 mL Eppendorf tube, and 1 mL of 80% methanol (*v*/*v*) was added. After 2-fold extraction, the extracts were subjected to a color reaction, and absorbance was measured using a FLUOstar OMEGA microplate reader. Total phenolic content was calculated based on a D-catechin calibration curve and expressed as mg/100 g of oil.

### 3.9. Statistical Analysis

All the analyses were performed in triplicate, and the values are reported in the tables as means ± standard deviations. Statistical analysis of the results was performed using Statistica 13.3 software (TIBCO, Palo Alto, CA, USA), which was employed for Tukey’s test for homogeneous groups, factorial analysis of variance (ANOVA), and principal component analysis (PCA). All the calculations were conducted at a significance level of *p* ≤ 0.05.

## 4. Conclusions

This study highlights the significant influence of cultivar type and cultivation system on the oil content and bioactive compound profile of two fiber-type linseed cultivars, Modran and Nike. “Cultivar” was decisive for seed oil, ALA, sterol, and phenolic compound contents. Continuous cropping of linseed was associated with increased carotenoid, phenol, and γ-tocopherol levels, suggesting that specific cultivation systems may enhance the production of targeted bioactive compounds. Although the continuous cropping system has been criticized for causing serious economic losses and other disadvantages related to crop quality, our results suggest that linseed oils cultivated using this practice have similar composition to oils from a crop rotation system. These findings highlight the potential to optimize cultivation practices to produce linseed oils with specific bioactive properties. 

Future research could aim to further refine these agronomic factors to maximize the bioactive profile of linseed oil, enhancing its value for applications in functional foods and nutraceuticals. Expanding the study to include diverse environmental conditions and agronomic practices, such as different soil types, irrigation methods, or fertilization regimes, could further refine our understanding of how these factors influence oil quality and yield. Additionally, conducting long-term studies across multiple years would help capture the effects of broader climatic trends and environmental variability on the properties of linseed oil. By addressing these aspects, future research could contribute to a more comprehensive framework for optimizing linseed cultivation for specific industrial or nutritional applications.

## Figures and Tables

**Table 1:** Oil content (% of seeds weight) and fatty acid composition (% of total fatty acids) of analyzed linseed oil samples.

No.	Year	Cultivar	Herbicide	Crop Rotation	Oil Yield	Fatty Acids					
						Palmitic (C16:0)	Stearic (C18:0)	Oleic (C18:1, n-9)	Vaccenic (C18:1, n-11)	Linoleic (C18:2)	α-Linolenic, ALA (C18:3, n-3)
1	2018	Nike	YES	YES	37.3 ± 0.0 ^ab^	4.92 ± 0.15 ^e^	4.81 ± 0.03 ^i^	21.2 ± 0.1 ^g^	0.495 ± 0.049 ^ab^	15.1 ± 0.1 ^a^	53.6 ± 0.2 ^a^
2	2018	Modran	YES	YES	37.2 ± 0.7 ^ab^	4.51 ± 0.03 ^abcd^	2.63 ± 0.01 ^bc^	17.4 ± 0.1 ^ef^	0.420 ± 0.028 ^ab^	15.1 ± 0.1 ^a^	59.9 ± 0.1 ^cde^
3	2018	Nike	YES	NO	38.1 ± 0.3 ^abcd^	4.80 ± 0.10 ^cde^	4.71 ± 0.01 ^hi^	20.6 ± 0.2 ^g^	0.490 ± 0.057 ^ab^	15.1 ± 0.2 ^a^	54.3 ± 0.2 ^a^
4	2018	Modran	YES	NO	35.4 ± 1.6 ^a^	4.52 ± 0.04 ^abcd^	2.63 ± 0.01 ^bc^	17.4 ± 0.0 ^ef^	0.430 ± 0.028 ^ab^	14.6 ± 0.1 ^a^	60.4 ± 0.1 ^cdef^
5	2019	Nike	YES	YES	42.7 ± 0.9 ^f^	4.47 ± 0.02 ^abcd^	3.28 ± 0.01 ^d^	15.5 ± 0.0 ^bc^	0.435 ± 0.007 ^ab^	17.2 ± 0.1 ^cd^	59.1 ± 0.0 ^bc^
6	2019	Modran	YES	YES	39.6 ± 0.2 ^bcde^	4.31 ± 0.03 ^a^	2.32 ± 0.02 ^a^	14.5 ± 0.1 ^ab^	0.385 ± 0.007 ^a^	16.8 ± 0.2 ^bcd^	61.7 ± 0.2 ^fg^
7	2019	Modran	YES	NO	42.5 ± 1.7 ^ef^	4.43 ± 0.01 ^abc^	2.50 ± 0.01 ^ab^	14.1 ± 0.1 ^a^	0.430 ± 0.014 ^ab^	16.3 ± 0.1 ^b^	62.2 ± 0.1 ^g^
8	2019	Nike	YES	NO	40.9 ± 0.1 ^def^	4.49 ± 0.05 ^abcd^	3.70 ± 0.04 ^ef^	14.3 ± 0.0 ^a^	0.425 ± 0.021 ^ab^	16.6 ± 0.1 ^bc^	60.5 ± 0.2 ^cdef^
9	2018	Modran	NO	NO	38.1 ± 0.3 ^abcd^	4.66 ± 0.25 ^abcde^	2.79 ± 0.04 ^c^	18.5 ± 0.6 ^f^	0.435 ± 0.049 ^ab^	14.7 ± 0.4 ^a^	59.0 ± 0.7 ^bc^
10	2018	Nike	NO	NO	38.0 ± 0.1 ^abcd^	4.85 ± 0.01 ^de^	4.58 ± 0.07 ^gh^	20.8 ± 0.5 ^g^	0.510 ± 0.014 ^b^	15.0 ± 0.1 ^a^	54.2 ± 0.7 ^a^
11	2018	Modran	NO	YES	38.0 ± 0.2 ^abc^	4.56 ± 0.01 ^abcde^	2.57 ± 0.08 ^bc^	16.3 ± 0.2 ^cd^	0.405 ± 0.021 ^ab^	15.2 ± 0.3 ^a^	61.0 ± 0.4 ^defg^
12	2018	Nike	NO	YES	37.5 ± 0.3 ^ab^	4.72 ± 0.03 ^bcde^	4.41 ± 0.11 ^g^	20.8 ± 0.5 ^g^	0.505 ± 0.007 ^b^	14.8 ± 0.1 ^a^	54.7 ± 0.7 ^a^
13	2019	Modran	NO	NO	39.6 ± 0.1 ^bcde^	4.48 ± 0.10 ^abcd^	2.66 ± 0.05 ^bc^	14.5 ± 0.2 ^ab^	0.435 ± 0.021 ^ab^	16.6 ± 0,1 ^bc^	61.3 ± 0.4 ^efg^
14	2019	Nike	NO	NO	41.8 ± 1.1 ^ef^	4.74 ± 0.04 ^bcde^	3.80 ± 0.04 ^f^	14.2 ± 0.4 ^a^	0.410 ± 0.000 ^ab^	17.5 ± 0.4 ^d^	59.4 ± 0.8 ^bcd^
15	2019	Modran	NO	YES	40.8 ± 0.2 ^cdef^	4.47 ± 0.15 ^abcd^	2.48 ± 0.10 ^ab^	14.7 ± 0.2 ^ab^	0.475 ± 0.035 ^ab^	17.0 ± 0.1 ^bcd^	60.9 ± 0.4 ^defg^
16	2019	Nike	NO	YES	39.9 ± 0.4 ^bcdef^	4.41 ± 0.09 ^ab^	3.58 ± 0.06 ^e^	16.9 ± 0.0 ^de^	0.430 ± 0.000 ^ab^	16.4 ± 0.1 ^b^	58.2 ± 0.1 ^b^
Mean					39.2	4.58	3.34	17.0	0.445	15.9	58.8
SD					2.1	0.18	0.89	2.7	0.038	1.0	2.9
CV (%)					5.3	3.84	26.77	15.6	8.548	6.3	5.0

a–i—Means in the same column for all variants followed by different letters are significantly different (*p* ≤ 0.05). Values are mean ± SD; SD—standard deviation; CV—coefficient of variance.

**Table 2:** Sterol content (mg/kg of oil) in analyzed linseed oil samples.

No.	Year	Cultivar	Herbicide	Crop rotation	Campesterol	Stigmasterol	β-Sitosterol	Isofucosterol	Cycloartenol	24-Methylene-cyclolanostanol	Total Sterols
1	2018	Nike	YES	YES	748 ± 1 ^bc^	208 ± 2 ^f^	1558 ± 47 ^bcde^	385 ± 2 ^abc^	1115 ± 24 ^ab^	216 ± 5 ^d^	4230 ± 79 ^bc^
2	2018	Modran	YES	YES	870 ± 29 ^e^	190 ± 4 ^cdef^	1582 ± 17 ^cde^	504 ± 36 ^def^	1237 ± 22 ^bcd^	388 ± 19 ^fg^	4771 ± 127 ^ef^
3	2018	Nike	YES	NO	792 ± 10 ^cd^	207 ± 3 ^ef^	1630 ± 37 ^de^	393 ± 13 ^abc^	1167 ± 0 ^abc^	238 ± 4 ^d^	4427 ± 59 ^cd^
4	2018	Modran	YES	NO	873 ± 2 ^e^	194 ± 11 ^cdef^	1627 ± 29 ^cde^	500 ± 20 ^def^	1284 ± 3 ^cde^	408 ± 11 ^g^	4886 ± 19 ^fg^
5	2019	Nike	YES	YES	616 ± 5 ^a^	151 ± 6 ^a^	1372 ± 44 ^a^	383 ± 4 ^abc^	1088 ± 3 ^a^	109 ± 6 ^a^	3720 ± 48 ^a^
6	2019	Modran	YES	YES	744 ± 24 ^b^	168 ± 11 ^ab^	1442 ± 32 ^ab^	525 ± 33 ^f^	1305 ± 100 ^cde^	279 ± 19 ^d^	4462 ± 18 ^cd^
7	2019	Modran	YES	NO	794 ± 3 ^cd^	185 ± 4 ^bcde^	1523 ± 26 ^bcd^	530 ± 1 ^f^	1384 ± 1 ^ef^	362 ± 1 ^ef^	4778 ± 24 ^ef^
8	2019	Nike	YES	NO	726 ± 13 ^b^	199 ± 0 ^def^	1535 ± 40 ^bcd^	439 ± 19 ^bcd^	1191 ± 66 ^abc^	143 ± 2 ^b^	4233 ± 140 ^bc^
9	2018	Modran	NO	NO	819 ± 0 ^d^	186 ± 9 ^bcdef^	1536 ± 42 ^bcd^	448 ± 14 ^cde^	1214 ± 22 ^abc^	366 ± 1 ^ef^	4569 ± 88 ^de^
10	2018	Nike	NO	NO	724 ± 5 ^b^	191 ± 2 ^cdef^	1518 ± 1 ^bcd^	349 ± 11 ^a^	1111 ± 4 ^ab^	213 ± 1 ^d^	4106 ± 4 ^b^
11	2018	Modran	NO	YES	895 ± 15 ^e^	206 ± 4 ^ef^	1675 ± 30 ^e^	554 ± 2 ^f^	1364 ± 45 ^def^	385 ± 4 ^fg^	5079 ± 97 ^g^
12	2018	Nike	NO	YES	725 ± 7 ^b^	191 ± 3 ^cdef^	1552 ± 48 ^bcde^	380 ± 8 ^ab^	1191 ± 11 ^abc^	229 ± 5 ^d^	4267 ± 76 ^bc^
13	2019	Modran	NO	NO	796 ± 6 ^d^	177 ± 2 ^bcd^	1527 ± 33 ^bcd^	505 ± 23 ^ef^	1444 ± 4 ^f^	392 ± 2 ^fg^	4842 ± 54 ^efg^
14	2019	Nike	NO	NO	708 ± 9 ^b^	192 ± 8 ^cdef^	1558 ± 37 ^bcde^	442 ± 1 ^bcde^	1292 ± 18 ^cde^	161 ± 3 ^b^	4353 ± 69 ^bcd^
15	2019	Modran	NO	YES	740 ± 8 ^b^	177 ± 0 ^bc^	1493 ± 20 ^abc^	520 ± 2 ^f^	1483 ± 29 f	343 ± 4 ^e^	4756 ± 59 ^ef^
16	2019	Nike	NO	YES	709 ± 4 ^b^	183 ± 2 ^bcd^	1540 ± 20 ^bcde^	425 ± 1 ^bc^	1289 ± 0 ^cde^	173 ± 0 ^b^	4319 ± 16 ^bcd^
Mean					767	188	1542	455	1260	275	4487
SD					73	15	72	65	118	102	351
CV (%)					9	8	5	14	9	37	8

a–g—Means in the same column for all variants followed by different letters are significantly different (*p* ≤ 0.05). Values are mean ± SD; SD—standard deviation; CV—coefficient of variance.

**Table 3:** Tocochromanol, carotenoid, and phenolic content (mg/kg of oil) in analyzed linseed oil samples.

No.	Year	Cultivar	Herbicide	Crop Rotation	Plastochromanol-8	γ-Tocopherol	Total Tocochromanols	Total Carotenoids	Total Phenolics
1	2018	Nike	YES	YES	219 ± 4 ^g^	459 ± 8 ^fg^	678 ± 12 ^hi^	16 ± 0 ^fg^	67 ± 4 ^a^
2	2018	Modran	YES	YES	173 ± 2 ^de^	454 ± 4 ^ef^	627 ± 6 ^def^	13 ± 0 ^de^	106 ± 0 ^abcde^
3	2018	Nike	YES	NO	206 ± 1 ^fg^	483 ± 4 ^h^	690 ± 2 ^i^	17 ± 0 ^g^	148 ± 1 ^cde^
4	2018	Modran	YES	NO	172 ± 2 ^de^	488 ± 9 ^h^	661 ± 7 ^gh^	15 ± 1 ^fg^	159 ± 40 ^e^
5	2019	Nike	YES	YES	162 ± 5 ^bcd^	408 ± 1 ^a^	570 ± 6 ^a^	9 ± 0 ^a^	72 ± 8 ^ab^
6	2019	Modran	YES	YES	156 ± 2 ^ab^	426 ± 0 ^abc^	582 ± 2 ^ab^	10 ± 0 ^ab^	88 ± 26 ^abcd^
7	2019	Modran	YES	NO	159 ± 2 ^abc^	447 ± 0 ^ef^	606 ± 2 ^bcd^	14 ± 0 ^ef^	60 ± 4 ^a^
8	2019	Nike	YES	NO	180 ± 1 ^e^	474 ± 5 ^gh^	654 ± 7 ^fgh^	11 ± 0 ^bcd^	63 ± 1 ^a^
9	2018	Modran	NO	NO	171 ± 4 ^cde^	457 ± 7 ^efg^	628 ± 11 ^def^	16 ± 0 ^g^	154 ± 41 ^de^
10	2018	Nike	NO	NO	200 ± 4 ^f^	479 ± 2 ^h^	679 ± 6 ^hi^	17 ± 0 ^g^	104 ± 4 ^abcde^
11	2018	Modran	NO	YES	147 ± 3 ^a^	418 ± 5 ^ab^	565 ± 8 ^a^	15 ± 1 ^fg^	119 ± 16 ^abcde^
12	2018	Nike	NO	YES	193 ± 5 ^f^	445 ± 3 ^def^	638 ± 8 ^efg^	14 ± 1 ^ef^	83 ± 3 ^abc^
13	2019	Modran	NO	NO	166 ± 1 ^bcd^	446 ± 7 ^ef^	612 ± 7 ^cde^	12 ± 0 ^cde^	136 ± 12 ^bcde^
14	2019	Nike	NO	NO	175 ± 6 ^de^	439 ± 0 ^cde^	614 ± 6 ^cde^	10 ± 0 ^abc^	106 ± 3 ^abcde^
15	2019	Modran	NO	YES	155 ± 1 ^ab^	408 ± 1 ^ab^	564 ± 2 ^a^	10 ± 0 ^abc^	53 ± 1 ^a^
16	2019	Nike	NO	YES	171 ± 3 ^cde^	427 ± 5 ^bcd^	598 ± 7 ^bc^	11 ± 1 ^bcd^	58 ± 1 ^a^
Mean					175	447	623	13	98
SD					20	26	41	3	36
CV (%)					11	6	7	21	37

a–i—Means in the same column for all variants followed by different letters are significantly different (*p* ≤ 0.05). Values are mean ± SD; SD—standard deviation; CV—coefficient of variance.

**Table 4:** Correlation matrix of analyzed variables.

	Oil Content	Palmitic Acid	Stearic Acid	Oleic Acid	Vaccenic Acid	Linoleic Acid	ALA	Campesterol	Stigmasterol	ß-Sitosterol	Isofucosterol	Cycloartenol	24-Methylene-cyclolanostanol	Total Sterols	Total Carotenoids	Total Phenols	Plastochromanol-8	γ -Tocopherol	Total Tocochromanols
Oil content	-	−0.40	−0.18	−0.70 *	−0.30	0.89 *	0.41	−0.63 *	−0.56 *	−0.63 *	0.06	0.21	−0.43	−0.33	−0.68 *	−0.57 *	−0.41	−0.55 *	−0.54 *
Palmitic acid		-	0.81 *	0.78 *	0.71 *	−0.50 *	−0.85 *	−0.03	0.61 *	0.36	−0.67 *	−0.56 *	−0.24	−0.29	0.65*	0.25	0.83 *	0.49	0.70 *
Stearic acid		*	-	0.71 *	0.73 *	−0.25	−0.92 *	−0.42	0.45	0.13	−0.89 *	−0.71 *	−0.66 *	−0.64 *	0.35	−0.09	0.91 *	0.43	0.70 *
Oleic acid	*	*	*	-	0.77 *	−0.79 *	−0.91 *	0.08	0.48	0.34	−0.66 *	−0.64 *	−0.10	−0.26	0.73*	0.26	0.81 *	0.50	0.70 *
Vaccenic acid		*	*	*	-	−0.42	−0.83 *	−0.24	0.29	0.06	−0.68 *	−0.40	−0.21	−0.35	0.49	−0.08	0.75 *	0.34	0.57 *
Linoleic acid	*	*		*		-	0.49	−0.62 *	−0.60 *	−0.62 *	0.17	0.34	−0.41	−0.25	−0.87 *	−0.52 *	−0.46	−0.60 *	−0.59 *
ALA		*	*	*	*		-	0.27	−0.41	−0.15	0.86 *	0.72 *	0.45	0.54 *	−0.51 *	−0.04	−0.91 *	−0.41	−0.69 *
Campesterol	*					*		-	0.52 *	0.77 *	0.61 *	0.37	0.86 *	0.88 *	0.56 *	0.60 *	−0.18	0.33	0.12
Stigmasterol	*	*				*		*	-	0.87 *	−0.09	−0.13	0.13	0.31	0.70 *	0.27	0.53 *	0.61 *	0.63 *
ß-Sitosterol	*					*		*	*	-	0.20	0.14	0.43	0.61 *	0.64 *	0.51 *	0.21	0.47	0.39
Isofucosterol		*	*	*	*		*	*			-	0.83 *	0.74 *	0.85 *	−0.20	0.10	−0.79 *	−0.32	−0.58 *
Cycloartenol		*	*	*			*				*	-	0.62 *	0.77 *	−0.25	−0.02	−0.69 *	−0.40	−0.58 *
24-Methylene-cyclolanostanol			*					*			*	*	-	0.91 *	0.37	0.49	−0.40	0.08	−0.15
Total sterols			*				*	*		*	*	*	*	-	0.26	0.39	−0.47	0.02	−0.21
Total carotenoids	*	*		*		*	*	*	*	*					-	0.55 *	0.52 *	0.68 *	0.67 *
Total phenols	*					*		*		*					*	-	0.06	0.50	0.34
Plastochromanol-8		*	*	*	*		*		*		*	*			*		-	0.65 *	0.88 *
γ-Tocopherol	*					*			*						*		*	-	0.93 *
Total tocochromanols	*	*	*	*	*	*	*		*		*	*			*		*	*	-

*—correlation statistically significant at *p* ≤ 0.05; the greener the color, the stronger the positive correlation; the redder the color, the stronger the negative correlation; ALA—α-linolenic acid.

**Table 5:** Atmospheric precipitation and daily air temperature during the study period (2018–2019) according to the Meteorological Station in Bałcyny, Poland.

Year	Month						Sum/Mean III–VIII
	March	April	May	June	July	August	
Precipitation (mm)							
2018	25.0	28.1	41.0	64.7	140.7	31.2	330.7
2019	30.2	0.0	97.8	92.0	85.8	64.8	370.6
1991–2020	30.9	29	62.4	72.5	91.9	66.1	352.8
Air temperature (°C)							
2018	−0.5	11.9	16.5	17.9	20.0	20.4	14.4
2019	4.9	8.6	12.2	21.4	17.6	19.5	14.0
1991–2020	2.1	8.1	13.1	16.4	18.5	18.3	12.8

## Data Availability

The raw data supporting the conclusions of this article will be made available by the authors on request.

## Supplementary Materials

The following supporting information can be downloaded at: https://www.mdpi.com/article/10.3390/molecules30040875/s1. Table S1: Explanation of variance for determined values.

## References

[1] Dudarev I. (2022). A Review of Fibre Flax Harvesting: Conditions, Technologies, Processes and Machines. J. Nat. Fibers.

[2] FAOSTAT Crops and Livestock Products. https://www.fao.org/faostat/en/#data/QCL/visualize.

[3] Al-Madhagy S., Ashmawy N.S., Mamdouh A., Eldahshan O.A., Farag M.A. (2023). A Comprehensive Review of the Health Benefits of Flaxseed Oil in Relation to Its Chemical Composition and Comparison with Other Omega-3-Rich Oils. Eur. J. Med. Res..

[4] Pramanik J., Kumar A., Prajapati B. (2023). A Review on Flaxseeds: Nutritional Profile, Health Benefits, Value Added Products, and Toxicity. eFood.

[5] Isanejad M., Tajik B., McArdle A., Tuppurainen M., Sirola J., Kröger H., Rikkonen T., Erkkilä A. (2022). Dietary Omega-3 Polyunsaturated Fatty Acid and Alpha-Linolenic Acid Are Associated with Physical Capacity Measure but Not Muscle Mass in Older Women 65–72 Years. Eur. J. Nutr..

[6] Dąbrowski G., Konopka I., Czaplicki S. (2018). Supercritical CO_2_ Extraction in Chia Oils Production: Impact of Process Duration and Co-Solvent Addition. Food Sci. Biotechnol..

[7] Kulczyński B., Kobus-Cisowska J., Taczanowski M., Kmiecik D., Gramza-Michałowska A. (2019). The Chemical Composition and Nutritional Value of Chia Seeds—Current State of Knowledge. Nutrients.

[8] Rizzo G., Baroni L., Lombardo M. (2023). Promising Sources of Plant-Derived Polyunsaturated Fatty Acids: A Narrative Review. Int. J. Environ. Res. Public Health.

[9] Pali V., Mehta N. (2014). Evaluation of Oil Content and Fatty Acid Compositions of Flax (*Linum usitatissimum* L.) Varieties of India. J. Agr. Sci..

[10] Silska G., Walkowiak M. (2019). Comparative Analysis of Fatty Acid Composition in 84 Accessions of Flax (*Linum usitatissimum* L.). J. Pre. Clin. Clin. Res..

[11] Bayrak A., Kiralan M., Ipek A., Arslan N., Cosge B., Khawar K.M. (2010). Fatty Acid Compositions of Linseed (*Linum usitatissimum* L.) Genotypes of Different Origin Cultivated in Turkey. Biotechnol. Biotec. Eq..

[12] Angove O. (2021). Call for an Oilseeds Crop Diversification in Finland. Bachelor’s Thesis.

[13] Kauser S., Hussain A., Ashraf S., Fatima G., Javaria S., Abideen Z.U., Kabir K., Yaqub S., Akram S., Shehzad A. (2024). Flaxseed (*Linum usitatissimum*); Phytochemistry, Pharmacological Characteristics and Functional Food Applications. Food Chem. Adv..

[14] Rossi A., Clemente C., Tavarini S., Angelini L.G. (2022). Variety and Sowing Date Affect Seed Yield and Chemical Composition of Linseed Grown under Organic Production System in a Semiarid Mediterranean Environment. Agronomy.

[15] Čeh B., Štraus S., Hladnik A., Kušar A. (2020). Impact of Lnseed Variety, Location and Production Year on Seed Yield, Oil Content and Its Composition. Agronomy.

[16] Stavropoulos P., Mavroeidis A., Papadopoulos G., Roussis I., Bilalis D., Kakabouki I. (2023). On the Path towards a “Greener” EU: A Mini Review on Flax (*Linum usitatissimum* L.) as a Case Study. Plants.

[17] Anastasiu A.-E., Chira N.-A., Banu I., Ionescu N., Stan R., Rosca S.-I. (2016). Oil Productivity of Seven Romanian Linseed Varieties as Affected by Weather Conditions. Ind. Crops Prod..

[18] (2023). Policies for the Future of Farming and Food in the European Union.

[19] Jastrzębska M., Kostrzewska M.K., Marks M. (2024). Over 50 Years of a Field Experiment on Cropping Systems in Bałcyny, Poland: Assessing Pesticide Residues in Soil and Crops from the Perspective of Their Field Application History. Eur. J. Agron..

[20] Johnston A.E., Poulton P.R. (2018). The Importance of Long-term Experiments in Agriculture: Their Management to Ensure Continued Crop Production and Soil Fertility; the Rothamsted Experience. Eur. J. Soil Sci..

[21] Kostrzewska M.K., Jastrzębska M. (2024). Exploiting the Yield Potential of Spring Barley in Poland: The Roles of Crop Rotation, Cultivar, and Plant Protection. Agriculture.

[22] Filipovic V., Popovic D., Glamoclija D., Jaramaz M., Jaramaz D., Andelovic S., Tabakovic M. (2014). Genotype and Soil Type Influence on Morphological Characteristics, Yield and Oil Content of Oil-Flax. Bulg. J. Agric. Sci..

[23] Jarošová M., Lorenc F., Bedrníček J., Petrášková E., Bjelková M., Bártová V., Jarošová E., Zdráhal Z., Kyselka J., Smetana P. (2024). Comparison of Yield Characteristics, Chemical Composition, Lignans Content and Antioxidant Potential of Experimentally Grown Six Linseed (*Linum usitatissimum* L.) Cultivars. Plant Foods Hum. Nutr..

[24] Danish M., Nizami M. (2019). Complete Fatty Acid Analysis Data of Flaxseed Oil Using GC-FID Method. Data Brief.

[25] Goyal A., Sharma V., Upadhyay N., Gill S., Sihag M. (2014). Flax and Flaxseed Oil: An Ancient Medicine & Modern Functional Food. J. Food Sci. Technol..

[26] Trela A., Silska G., Chyc M., Latowski D., Kruk J., Szymańska R. (2019). Tocochromanols and Fatty Acid Composition in Flax (*Linum usitatissimum* L.) Accessions. Acta Soc. Bot. Pol..

[27] Walkowiak M., Spasibionek S., Krótka K. (2022). Variation and Genetic Analysis of Fatty Acid Composition in Flax (*Linum usitatissimum* L.). Euphytica.

[28] Klein J., Zikeli S., Claupein W., Gruber S. (2017). Linseed (*Linum usitatissimum*) as an Oil Crop in Organic Farming: Abiotic Impacts on Seed Ingredients and Yield. Org. Agric..

[29] Andruszczak S., Gawlik-Dziki U., Kraska P., Kwiecińska-Poppe E., Różyło K., Pałys E. (2015). Yield and Quality Traits of Two Linseed (*Linum usitatissimum* L.) Cultivars as Affected by Some Agronomic Factors. Plant Soil Environ..

[30] Ciftci O.N., Przybylski R., Rudzińska M. (2012). Lipid Components of Flax, Perilla, and Chia Seeds. Eur. J. Lipid Sci. Tech..

[31] Teneva O., Zlatanov M.D., Antova G.A., Angelova-Romova M., Marcheva M. (2013). Lipid Composition of Flaxseeds. Bulg. Chem. Commun..

[32] Gandova V., Teneva O., Petkova Z., Iliev I., Stoyanova A. (2023). Lipid Composition and Physicochemical Parameters of Flaxseed Oil (*Linum usitatissimum* L.) from Bulgaria. Appl. Sci..

[33] Herchi W., Harrabi S., Sebei K., Rochut S., Boukhchina S., Pepe C., Kallel H. (2009). Phytosterols Accumulation in the Seeds of *Linum usitatissimum* L. Plant Physiol. Bioch..

[34] Gębarowski T., Wiatrak B., Jęśkowiak-Kossakowska I., Grajzer M., Prescha A. (2023). Oils from Transgenic Flax Lines as Potential Chemopreventive Agents in Colorectal Cancer. Biomedicines.

[35] Obranović M., Škevin D., Kraljić K., Pospišil M., Neđeral S., Blekić M., Putnik P. (2015). Influence of Climate, Varieties and Production Process on Tocopherols, Plastochromanol-8 and Pigments in Flaxseed Oil. Food Technol. Biotechnol..

[36] Hasiewicz-Derkacz K., Kulma A., Czuj T., Prescha A., Żuk M., Grajzer M., Łukaszewicz M., Szopa J. (2015). Natural Phenolics Greatly Increase Flax (*Linum usitatissimum*) Oil Stability. BMC Biotechnol..

[37] Tavarini S., Castagna A., Conte G., Foschi L., Sanmartin C., Incrocci L., Ranieri A., Serra A., Angelini L.G. (2019). Evaluation of Chemical Composition of Two Linseed Varieties as Sources of Health-Beneficial Substances. Molecules.

[38] Herchi W., Sakouhi F., Arráez-Román D., Segura-Carretero A., Boukhchina S., Kallel H., Fernández-Gutierrez A. (2011). Changes in the Content of Phenolic Compounds in Flaxseed Oil During Development. J. Am. Oil Chem. Soc..

[39] Özcan M.M., Uslu N. (2022). Investigation of Changes in Some Chemical Properties, Bioactive Compounds, Antioxidant Activity, Phenolic and Fatty Acid Profiles of Flaxseed and Oils. J. Food Process. Preserv..

[40] Zhang X., Zhang Y., Sun P., Su W., Qu Z., Dong Y., Du S., Yu X. (2023). Effect of Germination Pretreatment on the Physicochemical Properties and Lipid Concomitants of Flaxseed Oil. RSC Adv..

[41] (2013). Oilseed Meals—Determination of Oil Content (Reference Method).

[42] Dąbrowski G., Konopka I., Czaplicki S. (2018). Variation in Oil Quality and Content of Low Molecular Lipophilic Compounds in Chia Seed Oils. Int. J. Food Prop..

[43] Toro-Vazquez J.F. (1991). Interactions among Oil Components during Adsorption: Effects on Carotenoids and Peroxides. J. Food Sci..

[44] Siger A., Nogala-Kałucka M., Lampart-Szczapa E. (2008). The Content and Antioxidant Activity of Phenolic Compounds in Cold-pressed Plant Oils. J. Food Lipids.

