# Supplementary material for: Variation in Linseed Oil Composition: Impact of Cultivar, Cultivation System, and Year of Cultivation
Source: Molecules. 2025 Feb 14;30(4):875. doi: 10.3390/molecules30040875 (PMC11858308; doi:10.3390/molecules30040875)
Supplement: Supplementary file 1 [file molecules-30-00875-s001.zip › molecules-3413629-supplementary.pdf]

Table S1. Explanation of variance for determined values.

Effect	Degree of free-dom	oil content				ALA (C18:3, n-3)				ß-sitosterol				total sterols			
		SS	MS	F	p	SS	MS	F	p	SS	MS	F	p	SS	MS	F	p
y-intercept	1	49236	49236	94626	0.0000*	110580	110580	638767	0.0000*	76062355	76062355	66390.77	0.0000*	6443842450	644384250	123738	0.0000*
Year	1	98.3	98.3	189	0.0000*	86.8	86.8	501	0.0000*	58948	58948	51.45	0.0000*	95010	95010	18.2	0.0006*
Cultivar	1	3.17	3.17	6.10	0.0252*	132	132	760	0.0000*	2535	2535	2.21	0.1563	2518249	2518249	484	0.0000*
Herbicide	1	0.00	0.00	0.00	1.0000	1.0	1.0	5.8	0.0284*	2060	2060	1.80	0.1987	76788	76788	14.7	0.0014*
Crop rotation	1	0.27	0.27	0.52	0.4819	0.6	0.6	3.4	0.0844	7232	7232	6.31	0.0231*	43606	43606	8.4	0.0106*
Year*Cultivar	1	0.06	0.06	0.12	0.7345	26.4	26.4	152	0.0000*	4077	4077	3.56	0.0775	457	457	0.1	0.7708
Year*Herbicide	1	6.43	6.43	12.35	0.0029*	2.40	2.40	13.9	0.0018*	16268	16268	14.20	0.0017*	234415	234415	45.0	0.0000*
Cultivar*Herbi-cide	1	1.67	1.67	3.21	0.0919	0.200	0.200	1.00	0.3369	29	29	0.03	0.8755	947	947	0.2	0.6755
Year*Crop rota-tion	1	0.58	0.58	1.12	0.3056	3.0	3.0	17.4	0.0007*	15394	15394	13.44	0.0021*	214107	214107	41.1	0.0000*
Cultivar*Crop rotation	1	0.19	0.19	0.37	0.5524	1.6	1.6	9.1	0.0083*	4896	4896	4.27	0.0553	41681	41681	8.0	0.0121*
Herbicide*Crop rotation	1	0.28	0.28	0.53	0.4754	2.1	2.1	12.3	0.0029*	28817	28817	25.15	0.0001*	357361	357361	68.6	0.0000*
Year*Culti-var*Herbicide	1	1.17	1.17	2.25	0.1533	0.3	0.3	1.8	0.2022	8192	8192	7.15	0.0166*	50435	50435	9.7	0.0067*
Year*Culti-var*Crop rota-tion	1	2.59	2.59	4.98	0.0403*	0.0	0.0	0.0	0.9433	543	543	0.47	0.5009	10233	10233	2.0	0.1801
Year*Herbi-cide*Crop rota-tion	1	0.48	0.48	0.93	0.3488	1.5	1.5	8.6	0.0099*	1197	1197	1.04	0.3219	9456	9456	1.8	0.1966
Cultivar*Herbi-cide*Crop rota-tion	1	3.89	3.89	7.47	0.0147*	0.2	0.2	1.0	0.3329	47	47	0.04	0.8421	34	34	0.0	0.9364
Year*Culti-var*Herbi-cide*Crop rota-tion	1	12.12	12.12	23.29	0.0002*	0.2	0.2	1.3	0.2665	3799	3799	3.32	0.0874	33125	33125	6.4	0.0226*
Error	16	8.33	0.52			2.8	0.2			18331	1146			83322	5208		
Total	31	139				261				172366				3769226			

Effect	Degree of freedom	plastochromanol-8				γ-tocopherol				total carotenoids				total phenolics			
		SS	MS	F	p	SS	MS	F	p	SS	MS	F	p	SS	MS	F	P
y-intercept	1	984923	984923	92276	0.0000*	6404815	6404815	291387	0.0000*	5497	5497	16895	0.0000*	1241340	1241340	1103	0.0000*
Year	1	3136	3136	294	0.0000*	5447	5447	247.8	0.0000*	148	148	455	0.0000*	46124	46124	41.0	0.0000*
Cultivar	1	5425	5425	508	0.0000*	618	618	28.1	0.0001*	0.348	0.348	1.07	0.3167	15216	15216	13.5	0.0020*
Herbicide	1	312	312	29.3	0.0001*	1779	1779	80.9	0.0000*	1.15	1.15	3.54	0.0782	1295	1295	1.15	0.2994
Crop rotation	1	345	345	32.3	0.0000*	8970	8970	408.1	0.0000*	33.1	33.1	102	0.0000*	40286	40286	35.80	0.0000*
Year*Cultivar	1	1350	1350	126	0.0000*	97	97	4.4	0.0517	9.65	9.65	29.7	0.0001*	4939	4939	4.39	0.0524
Year*Herbicide	1	569	569	53.3	0.0000*	358	358	16.3	0.0010*	0.310	0.310	0.95	0.3434	3891	3891	3.46	0.0814
Cultivar*Herbicide	1	4.4	4.4	0.41	0.5292	335	335	15.2	0.0013*	0.773	0.773	2.38	0.1428	1139	1139	1.01	0.3293
Year*Crop rotation	1	34.4	34.4	3.22	0.0916	4	4	0.2	0.6898	0.003	0.003	0.01	0.9194	4700	4700	4.18	0.0578
Cultivar*Crop rotation	1	54.1	54.1	5.07	0.0388*	4	4	0.2	0.6921	2.21	2.206	6.78	0.0192*	3	3	0.003	0.9597
Herbicide*Crop rotation	1	178	178	16.7	0.0009*	68	68	3.1	0.0976	2.53	2.531	7.78	0.0131*	3993	3993	3.55	0.0779
Year*Cultivar*Herbicide	1	0.9	0.9	0.08	0.7757	270	270	12.3	0.0030*	7.94	7.944	24.4	0.0001*	338	338	0.301	0.5910
Year*Cultivar*Crop rotation	1	176	176	16.5	0.0009*	140	140	6.3	0.0227*	3.10	3.10	9.52	0.0071*	469	469	0.416	0.5279
Year*Herbicide*Crop rotation	1	343	343	32.1	0.0000*	323	323	14.7	0.0015*	5.45	5.45	16.8	0.0008*	30186	30186	26.8	0.0001*
Cultivar*Herbicide*Crop rotation	1	102	102	9.56	0.0070*	516	516	23.5	0.0002*	0.427	0.427	1.31	0.2687	4568	4568	4.06	0.0611
Year*Cultivar*Herbicide*Crop rotation	1	36.3	36.3	3.40	0.0837	712	712	32.4	0.0000*	2.775	2.775	8.53	0.0100*	56	56	0.050	0.8266
Error	16	170.8	10.7			352	22			5.206	0.325			18003	1125		
Total	31	12237				19992				223				175204			

Effects with "*" in "p" column are statistically significant at $p \leq 0.05$
